# Nb-Doped MXene With Enhanced Energy Storage Capacity and Stability

**DOI:** 10.3389/fchem.2020.00168

**Published:** 2020-04-03

**Authors:** Mahjabeen Fatima, Jameela Fatheema, Nasbah B. Monir, Ahmad Hassan Siddique, Bushra Khan, Amjad Islam, Deji Akinwande, Syed Rizwan

**Affiliations:** ^1^Physics Characterization and Simulations Lab (PCSL), School of Natural Sciences (SNS), National University of Sciences & Technology (NUST), Islamabad, Pakistan; ^2^Key Laboratory of Graphene Technologies and Applications of Zhejiang Province, Ningbo Institute of Materials Technology and Engineering (NIMTE), Ningbo, China; ^3^College of Materials Engineering, Fujian Agriculture and Forestry University, Fuzhou, China; ^4^Microelectronics Research Center, The University of Texas at Austin, Austin, TX, United States

**Keywords:** MXene, Nb doped MXene, 2D titanium carbide, supercapacitors, density functional theory

## Abstract

MXenes present unique features as materials for energy storage; however, limited interlayer distance, and structural stability with ongoing cycling limit their applications. Here, we have developed a unique method involving incorporating Nb atoms into MXene (Ti_3_C_2_) to enhance its ability to achieve higher ionic storage and longer stability. Computational analysis using density functional theory was performed that explained the material structure, electronic structure, band structure, and density of states in atomistic detail. Nb-doped MXene showed a good charge storage capacity of 442.7 F/g, which makes it applicable in a supercapacitor. X-ray diffraction (XRD) indicated c-lattice parameter enhancement after Nb-doping in MXene (from 19.2A° to 23.4A°), which showed the effect of the introduction of an element with a larger ionic radius (Nb). Also, the bandgap changes from 0.9 eV for pristine MXene to 0.1 eV for Nb-doped MXene, which indicates that the latter has the signature of increased conductivity due to more metallic nature, in support of the experimental results. This work presents not only the effect of doping in MXene but also helps to explain the phenomena involved in changes in physical parameters, advancing the field of energy storage based on 2D materials.

## Introduction

In the last decade, chemical processes have been developed to successfully produce a versatile family of two-dimensional (2D) transition metal carbides, nitrides, and carbonitrides, termed MXenes, from their parent layered MAX phases (Lei et al., 2015). Additionally, some unique and favorable properties of MXene have been discovered, creating tremendous opportunities for research and development, with potential applications in various fields such as energy storage devices, magnetic sensors, optoelectronic devices, etc. (Novoselov et al., 2004; Mariano et al., 2016; Zhuang et al., 2018; Sunaina et al., 2019). To date, more than 70 MAX phases have been reported, but the most popular and well-established of them include Ti_3_AlC_2_, Ti_2_AlC, (Ti_0.5_, Nb_0.5_)_2_C, (V_0.5_, Cr_0.5_)_3_C_2_, Ti_3_CN, Ta_4_AlC_3_, Nb_2_AlC, V_2_AlC, and Nb_4_AlC_3_ (Geim and Novoselov, 2007; Guo and Dong, 2011; Naguib et al., 2011a, 2012, 2013; Singh et al., 2011; Kuila et al., 2012; Tang and Zhou, 2013; Jing et al., 2014; Naguib and Gogotsi, 2015). The MAX titanium aluminum carbide is a layered, hexagonal carbide, or nitride form of compound, having the general formula “M_n+1_AX_n_”, where *n* = 1, 2, 3 and where M is a transition metal, A is an A-group element, and X represents carbon or nitrogen or a combination of both (Shein and Ivanovskii, 2013). Titanium aluminum carbide is used to make a daughter compound, i.e., MXene (Ti_3_C_2_), by eliminating aluminum from the layers of MAX to create paths for conduction of electrons along two dimensions. Notably, the outer surfaces of the exfoliated layers of MXene are always terminated by F, OH, and/or O groups during the chemical etching process (Hu et al., 2014). Henceforth, the terminated MXene species will be referred to as M_n+1_X_n_T_x_, where T represents the surface groups (F, OH, and/or O) and “x” is the number of terminations (Naguib et al., 2011b).

MXene, having higher conductivity compared to graphene, is advantageous for usage in the charging/discharging process in batteries (Nowak and Ziolek, 1999). MXene is preferred over graphene because of its better and modified properties, such as high specific surface area, electronic structure, and properties that differ from the bulk counterparts due to low dimensionality, i.e., a 2D lamellar structure, but also due to possessing hydrophilic surface properties and good electrical conductivity (Tang et al., 2018). Hence, MXene can be considered the best known 2D material for energy storage purposes.

Doping is performed to introduce impurities into 2D materials in order to induce desirable properties in the target material. After doping, many effects, such as an increase in the c-lattice parameter (c-LP) and the introduction of defects and impurities, can be obtained, which is useful for energy storage applications. Niobium (Nb) belongs to the fifth group of transition metals, with the oxidation state of +5 and a partially filled d-orbital. The incomplete d-orbital contributes to the stronger metal–metal bonding in the bulk material. It has a comparable ionic size to that of titanium (Ti; the ionic size of Nb has been reported to be 0.098 nm, while that of Ti is 0.097 nm), which makes it a suitable candidate to dope into Ti_3_C_2_ MXene (Tariq et al., 2018; Dashuai et al., 2019). Nb is highly reactive, which is useful in helping it attached to the surface of MXene. This article reports on an experimental and computational study of the effect of Nb-doping of Ti_3_C_2_ MXene from structural and morphological perspectives and for energy storage applications, such as in supercapacitors and lithium-ion batteries. This study helps reveal the possibility of increasing electronic conductivity in batteries by using doped MXene.

The main objective was to study the trend of MXene when doped with a transition metal, i.e., Nb, in order to observe the effects on the bandgap, as for pristine MXene, the bandgap was calculated to be 1.2 eV, which changed to 0.1 eV when doped with Nb.

## Experiment and Methods

MXene was formed from the aluminum etching of MAX Compound (Ti_3_AlC_2_). First, 10 g of MAX was dissolved in 230 ml of Hydrofluoric acid (HF, 39% wt%) in a Teflon beaker under constant magnetic stirring of 66 h at room temperature. After that time period, washing of MXene was performed by deionized water and ethanol several times to lower the pH of the obtained product. Washing was carried out in order to remove the acidic residues from MXene, and thus, after obtaining a pH of 6, vacuum filtration was performed to obtain the final product in the form of 2D layered MXene (Ti_3_C_2_), which was then dried in a vacuum oven for 16 h at 55°C.

For the preparation of Nb-doped MXene, etched MXene, i.e., Ti_3_C_2_, was treated with KMnO_4_ and ethylene glycol in order to secure MXene sheets from distortion. Then, 100 ml of Nb_2_O_5_ was prepared in 50 ml deionized water by introducing 25 ml ethyl glycol and 25 ml acetic acid into the deionized water at a ratio of 1:1; 200 ml Ti_3_C_2_ powder was then dispersed in the 100 ml aqueous solution of Nb_2_O_5_ under magnetic stirring at room temperature for 6 h. Next, 100 ml of KMnO_4_ aqueous solution was slowly added to the above solution and stirred for 30 min. After the addition of KMnO_4_, the color of the solution changed from grayish to dark purple, and, by the end, it turned dark gray. The solution was then rinsed with ethanol and deionized water three times, and extraction of precipitate was performed by vacuum filtration. It was then dried in a vacuum at 80°C for 24 h to obtain the powdered form of the desired material.

For the X-Ray Diffraction (XRD) study of the samples, a Bruker D8 Advance system was used to observe and analyze the diffraction peaks as well as the crystallinity. Additionally, for the study of morphology and elemental analysis, energy dispersive spectroscopy (EDS) was performed with a TESCAN VEGA 3. Transmission electron microscopy (with a ThermoFisher Talos F200X at an operation voltage of 200 keV) was used for analysis of the Nb-doped MXene to obtain high-resolution images. Cyclic voltammeter measurements were carried out using a Gamry Interface 1000E potentiostat, using Ag/AgCl as the electrodes. Moreover, the theoretical study is based on density functional theory. For simulations, the WIEN2k package was used, which employs the Full Potential Linear Augmented Plane Wave method (FP-LAPW) (Wimmer et al., 1981; Blaha et al., 2001). Firstly, the structure of Ti_3_C_2_ was generated and optimized at 500 k-points (k-mesh 14 × 14 × 2) using the Generalized Gradient Approximation PBE as well as WC GGA (Perdew et al., 1996; Wu and Cohen, 2016). The ground state of the structure was achieved by fully relaxing the internal coordinates. Subsequently, the band structure, density of states, and electron density were calculated using the GGA-PBE of the relaxed structure. To better understand the experimental results and structure, the c-parameter was increased up to 19.2 Å, and the band structure, density of states, and electron density were calculated. The next step was to simulate and optimize the Nb-doped MXene, for which WC-GGA was used at 500 k-points. The electron density, density of states, and band structure were studied for both the ground state structure and the structure based on the experimental parameters, i.e., c = 23.4 Å using the GGA-PBE functional.

## Results and Discussion

**Figure 1:**
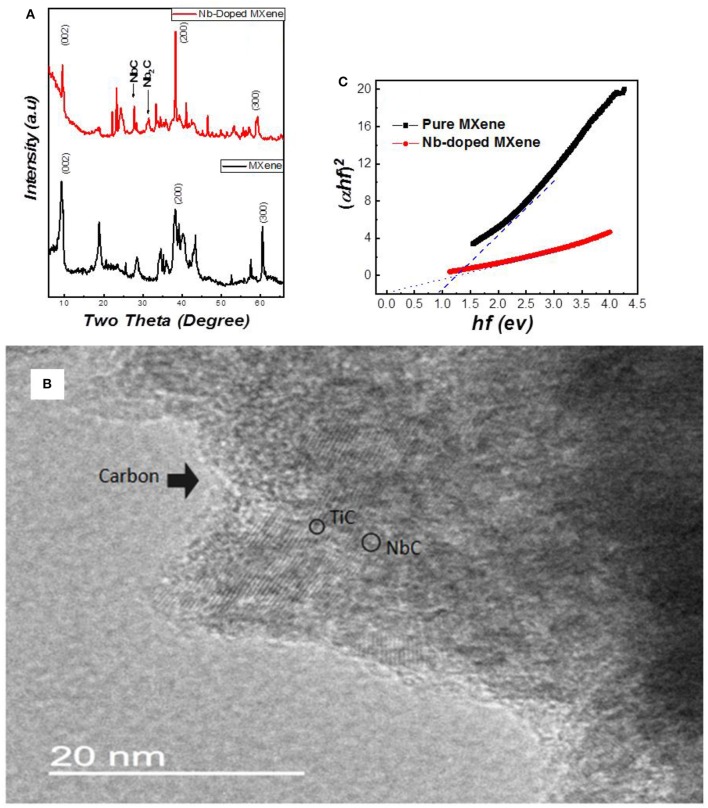
**(A)** X-ray diffraction of undoped (black) and Nb-doped (red) Ti_3_C_2_ MXene. **(B)** Transmission electron microscopy image of Nb-doped MXene. **(C)** Comparison of the bandgaps for undoped (black) and Nb-doped (red) Ti_3_C_2_ MXene.

Structural analysis of the Nb-doped sample was performed by X-ray diffractometer. The XRD graphs of MXene and Nb-doped MXene are shown in Figure 1A. It can be seen that the well-reported peaks of Ti_3_C_2_-MXene at 9, 18.8^0^, and 60.4^0^ are suppressed, with the appearance of new broader peaks. Due to the substitutional doping of Nb, two peaks appear at 28.5^0^ and 35.1^0^, which are referred to as niobium carbide (NbC, Nb_2_C) in the literature (Ivanovskii, 2007; Qing et al., 2012; Li et al., 2015, 2018; Su et al., 2018).

The peak appearing at 9.5^0^ in MXene is shifted to a lower angle, 9.3^0^. At 60^0^, there is a peak that is sharp and intense in the MXene but has decreased intensity in Nb-doped MXene, which is the result of the doping. In Figure 1B, carbon gives rough edges in the structure while the two contrasts i.e., light and dark pattern represents NbC and TiC respectively. Moreover Niobium replacing Titanium in MXene sheets gives Nb2CTx thin layers (Jia et al., 2011; Zhang et al., 2016). The presence of Nb in MXene has decreased the intensity of the MXene peak, which indicates that the amount of lattice defects has been increased (Tarascon and Armand, 2001). The peaks were analyzed analytically, and a comparison was made between the lattice parameters for undoped and doped samples. This shows that a-LP is 5.33A° for Ti_3_C_2_Tx and 5.28A° for Nb-Ti_3_C_2_, which is due to a slight ionic radii mismatch (the ionic radii of Nb and Ti are 0.069 nm and 0.068 nm, respectively). The value calculated for c-LP for Ti_3_C_2_ is 19.2A°, and that for Nb-Ti_3_C_2_ is 23.4A°, which shows intercalation between the MXene sheets.

Figure 1C represents the bandgaps of the samples as measured using UV-Vis spectroscopy through the absorbance and transmittance values of Nb-doped MXene. Like the MXene, Nb-doped MXene also shows a direct bandgap, as reported previously (Zifeng et al., 2016). The graphs obtained for Nb-doped MXene and MXene show that the bandgap has decreased from 0.9 eV to almost 0.1 eV for Nb-doped MXene. From EDX, it can be observed that substitutional doping of Nb occurred in Ti_3_C_2_ MXene (Figure S1). After doping, the Ti peaks become shorter with the increase in Nb peaks. Hence, we can confirm that Nb was successfully doped into the material both as a substitution of Ti and by penetration among the MXene sheets.

**Figure 2:**
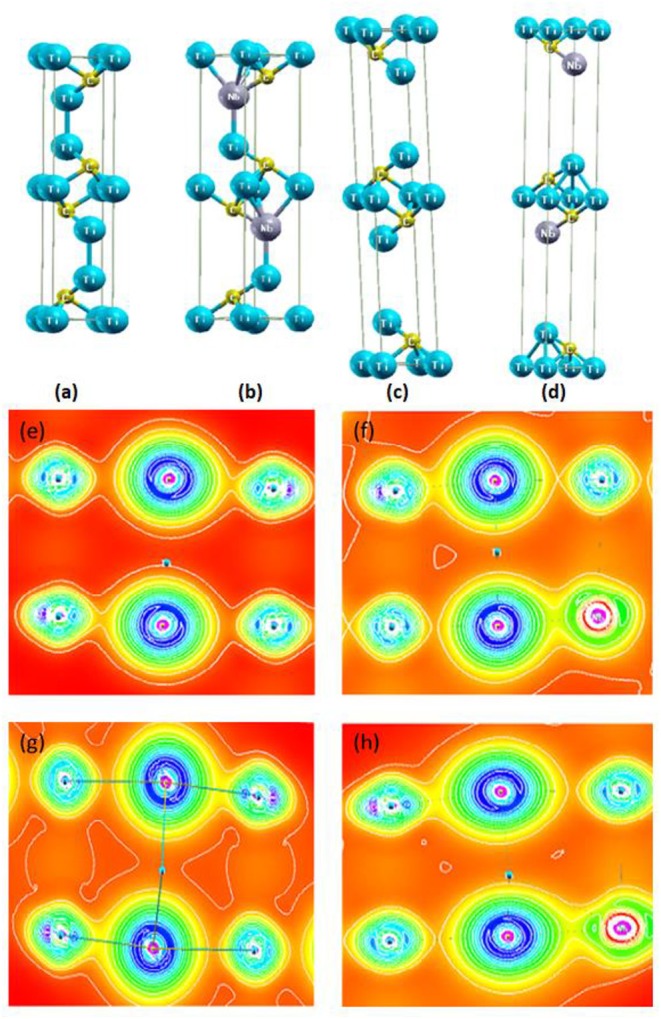
Structures of **(a)** Ti_3_C_2_, and **(b)** NbTi_2_C_2_ based on the relaxed parameter and structure of **(c)** Ti_3_C_2_, and **(d)** NbTi_2_C_2_ based on the experimental lattice parameter. Electron density of the relaxed structures for **(e)** Ti_3_C_2_, and **(f)** NbTi_2_C_2_. Electron density of the experimental structures for **(g)** Ti_3_C_2_, and **(h)** NbTi_2_C_2_.

For the ground-state properties, we relaxed the structure of Ti_3_C_2_ and NbTi_2_C_2_ by employing different exchange-correlation functionals in WIEN2k, as shown in Figures 2a,b. In Ti_3_C_2_, there are two non-equivalent Ti atoms, Ti1 and Ti2, where Ti1 occupies (1/3,2/3,u), (2/3,1/3,v) and Ti2 is present at (0,0,0) with space group P63/mmc. For the doped structure, Nb replaces Ti*1* at one of its Wyckoff positions.

The c-lattice parameters for relaxed structures of Ti_3_C_2_ and NbTi_2_C_2_ were found to be 15.0334 and 15.2018 Å, respectively, from the GGA-WC functional. The experimental and theoretical lattice parameters for Ti_3_C_2_ are different by almost 4 Å, whereas, for NbTi_2_C_2_, the difference is 8 Å. A similar situation is also reported by Naguib et al. who state that the reason for this is the instability of MXene surfaces in air, which causes bond formation of exposed Ti with oxygen (Naguib et al., 2011a). Furthermore, the reason for the huge difference in NbTi_2_C_2_ could be further reactions in MXene, i.e., while Nb enters between the sheets, it increases the c-parameter even more. The structures based on the experimental parameter are shown in Figures 2c,d.

The electronic properties of these structures were also studied computationally. Figures 2e,f shows the electron density of the relaxed Ti_3_C_2_ and NbTi_2_C_2_. The presence of the Nb atom changes the electron density and strengthens the bond, as Nb has a completely filled 3d shell and a partially filled 4d shell, while Ti only has three electrons in the 3d shell. In Figures 2g,h, when the lattice parameter is increased, we can see that the density becomes slightly different compared to the relaxed structures, as layers are now farther apart than before, and the area with the lowest electron density increases in size.

The band structure for pure MXene (Figure S2) shows a metallic behavior, since the bandgap is represented to be zero, whereas, from the UV-Visible Spectroscopy, we observed a bandgap of 0.9 eV. The reason for this mismatch in bandgap is the presence of termination groups on the surface. After doping with Nb, we observe that, around the Fermi level, there is an increase in the density of electrons. Also, the bandgap for the Nb-doped structure (Figure S2) remains zero, which is slightly more comparable to the experimental results.

For the structures with an enlarged c-parameter, we observe a slight difference in the band structure inside the valence band. For the purpose of pure MXene with relaxed parameters, a pink and violet band appears at −5.5 eV, and then, for enlarged parameters, it is shifted to −4.5eV (Figures S2A, S3A). In contrast, for the Nb-doped structure, this shift is from −6 to −4 eV (Figures S2B, S3B). Another point of difference is that the density of levels around Fermi levels is greater in the Nb-doped MXene than in the pure MXene. This is due to the electronic configuration of Nb, which has 10 electrons in the 3d orbital and then some more contribution from the electrons in the 4d orbital.

**Figure 3:**
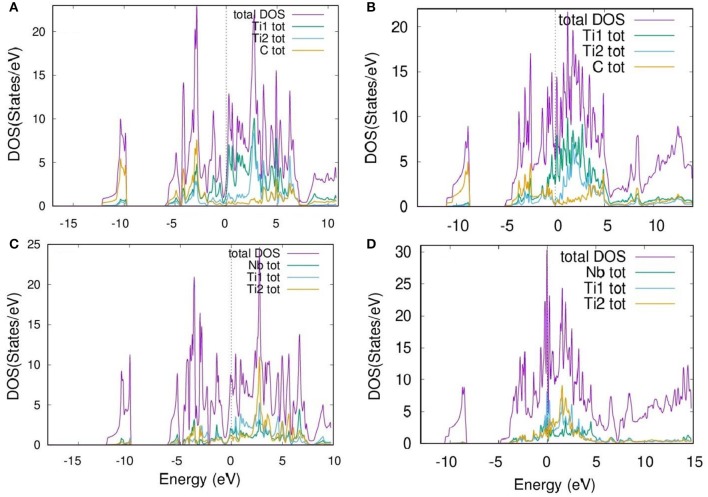
Density of states vs. energy plot for **(A)** Relaxed Ti_3_C_2_, **(B)** Experimental Ti_3_C_2_, **(C)** Relaxed NbTi_2_C_2_, and **(D)** Experimental NbTi_2_C_2_.

A density of states (DOS) vs. energy plot is presented in Figure 3, where Figures 3A,B are for relaxed and experimental Ti_3_C_2_, and Figures 3C,D are for relaxed and experimental NbTi_2_C_2_. The partial DOSs for the atoms and their corresponding shells are given in Figures S4, S5. From Figures 3A,B, we observe that the maximum value of DOS remains almost the same. The difference comes at the point where there is shifting of a few peaks from −5 eV to the Fermi level (0 eV), and similarly for the peaks at 5 eV. Figures S4A,B,D,E shows that the contribution from Ti1 and Ti2 is completely due to the d-orbital as that is the only valence orbital with free electrons. The difference in the DOS for Ti1 and Ti2 of relaxed parameters and experimental parameters was determined by the shift of peaks of the d-orbital. As the c-lattice parameter increases, the hybridization or bonding of one layer with the other layer decreases, resulting in more contribution of free electrons. For carbon (Figures S4C,D), the s-orbital is contributing mainly at the valence band, while the p-orbital is contributing in both the valence and conduction bands, showing the hybridization of the p-orbital with the d-orbital of Ti.

Furthermore, the Nb doping increases the density of states per eV. In the relaxed structure, it increases slightly (Figure 3C), but as the c-parameter is increased up to 23.4 A°, the DOS per state increases even more, especially around the Fermi energy level (Figure 3D). For the Nb atom, unlike the Ti atom, the contribution is not only from the d-orbital but a small amount of contribution comes also from the s-orbital, as the electronic configuration of Nb is [Kr] 4d^4^5s^1^. Besides that, for Ti1 and Ti2, the highest DOS peaks are at the Fermi energy level for given experimental parameters.

**Figure 4:**
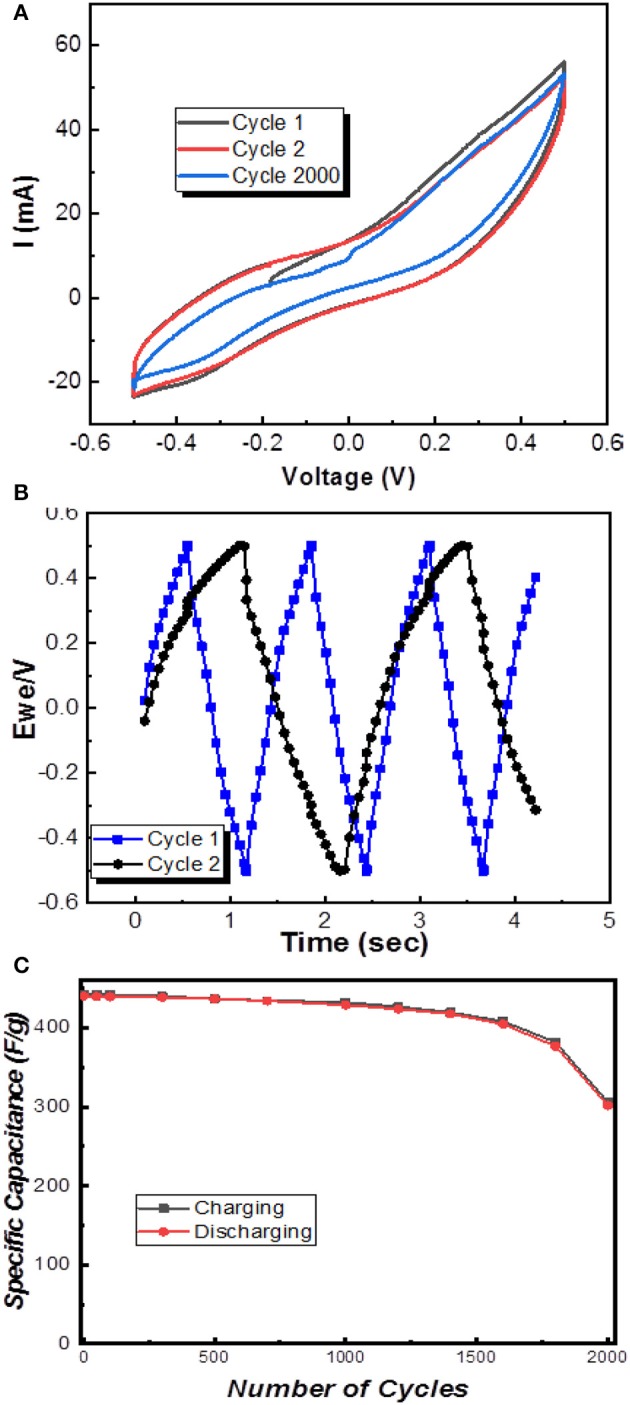
**(A)** Specific capacitance of Nb-doped MXene, **(B)** Charge-discharge curves of efficient cycle 1 and 2, and **(C)** Specific capacitance vs. number of cycles.

The cyclic voltammetry shows the I-V curve of Nb-doped MXene was observed at a potential window of −0.5V to +0.5V. From Figure 4A, the current vs. voltage graph enables the calculation of a maximum gravimetric capacitance of about 442.7 Farad/gram. This value of gravimetric capacitance of Nb-doped MXene sheets is about twice that reported for pure MXene previously (245 Farad/gram) in the literature (Li et al., 2017). The formula used was as follows:

(1) $$\begin{array}{l} C=\frac{Imax}{m\left(dv/dt\right)} \end{array}$$

To calculate storage capacity for charge, Equation (1) was considered, which shows that Nb-doped MXene exhibits high values of specific capacitance and enhanced performance of the MXene sheets and is inclusive of other factors such as higher specific area and enhanced storage ability because of its morphology of aligned flake-like structures (Zhao et al., 2015; Xiao et al., 2016). At higher current density, K^+^ ions, which are diffused from 6M KOH electrolyte, have abundant time to gain access and penetrate deep into the gaps available between different Nb-doped MXene layers easily, holding energetically, which leads to good a charge to discharge ratio (Chenhui et al., 2017; Ravuri et al., 2017). Also, the obtained charge-discharge triangular curves, which can be seen in Figure 4B, show excellent and enhanced electrode material performance with Nb-doping in MXene. Up to 1,000 cycles, the results obtained were excellent, as the last cycle's gravimetric capacitance was 432.5 F/grams. When it was further observed up to 1,500 cycles, the results were acceptable, but in the span from 1,600 to 2,000 cycles, the specific capacitance obtained was 306.8 F/gram. The charge/discharge curve values calculated for the first cycle were 442.7 and 440.5 F/g, respectively, whereas that for the discharge curve for 50 cycles was recorded to be 439.8 F/g and so on, as shown in Figure 4C, which shows a retaining trend in the specific capacitance and capability to exhibit stable and efficient electrode performance. Note that, in Figure 4A, cycle 1 and 2 denote the 1st cycle and 1000th cycle, whereas cycle 2,000 denotes the 2000th cycle as shown in Figure 4C. This directly reveals that this material can be applied in supercapacitors because of its very high specific capacitance as compared to some other two-dimensional materials like graphene; a comparison is presented in Table 1. Thus, we have proposed a material that can be used for supercapacitors.

**Table 1:** Comparison of specific capacitance of two-dimensional materials.

**Material**	**Charging Specific Capacitance**	**Electrolyte**
Graphene/Polyaniline Composite (Murugan et al., 2009)	408 F/g	1M H_2_SO_4_
Reduced Graphene Oxide/poly 3,4-ethylenedioxythiophene (Wen et al., 2014)	213 F/g	1M H_2_SO_4_
MXene (Ti3C2) (Cao et al., 2017)	124 F/g	KOH
Nb-doped MXene (Ti3C2) (current work)	442.7 F/g	6M KOH

## Conclusion

XRD analysis shows that, in addition to the peaks of MXene, we observed peaks of niobium carbide, niobium-doped MXene, and niobium-doped Ti_3_C_2_ MXene. Analytically, c-LP was calculated to show an increase from 19.2 to 23.4 Å. The bandgap of the prepared MXene was 0.9 eV, which decreases to 0.1 eV after being doped with Nb. Thus, the Nb-doped MXene can be considered a better conductor. In support of the experiment, computational analysis was also done that explained the electron structure, band structure, and density of states. It shows a maximum gravimetric capacitance of 442.7 Farad/gram, and the charge-discharge curves show excellent and enhanced performance for the electrode material. The present study hence suggests the unique behavior of the doped two-dimensional material bandgap, which is suitable for energy storage devices such as supercapacitors and lithium-ion batteries.

## Data Availability Statement

All datasets generated for this study are included in the article/Supplementary Material.

## Author Contributions

MF and NM prepared the samples and wrote the manuscript. JF worked on the density functional theory calculation. AS and AI helped in application characterization. BK helped in sample preparation. DA helped in manuscript writing, and SR conceived the research idea and supervised the whole project.

### Conflict of Interest

The authors declare that the research was conducted in the absence of any commercial or financial relationships that could be construed as a potential conflict of interest.
