# Supplementary material for: Nb-Doped MXene With Enhanced Energy Storage Capacity and Stability
Source: Front Chem. 2020 Apr 3;8:168. doi: 10.3389/fchem.2020.00168 (PMC7145951; doi:10.3389/fchem.2020.00168)
Supplement: Supplementary file 1 [file Table_1.DOCX]

**Nb-doped MXene with Enhanced Energy Storage Capacity and Stability**

Supplementary Information


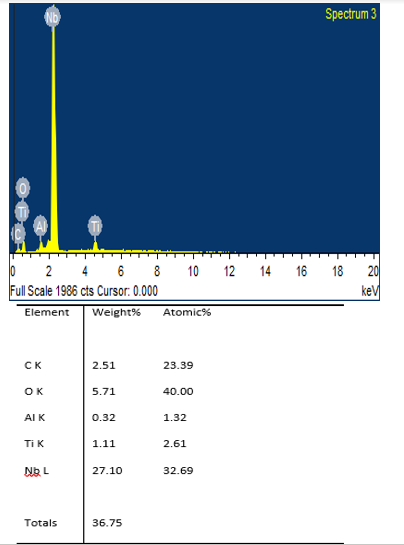


**Figure S1** EDX pattern of niobium-doped MXene


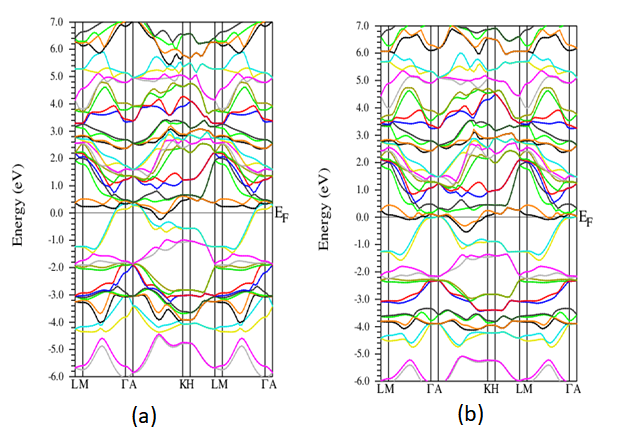


**Figure S2** (a) band structure of relaxed Ti_3_C_2_ and (b) band structure of relaxed NbTi_2_C_2_.


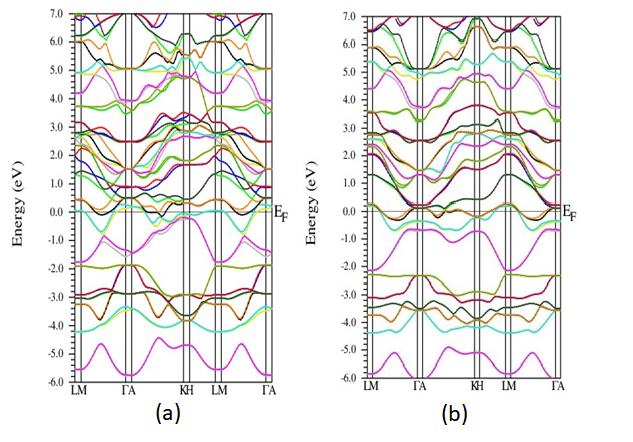


**Figure S3** (a) band structure of Ti_3_C_2_ with c=19.2A°, and (b) band structure of relaxed NbTi_2_C_2_ with c=23.4A°.


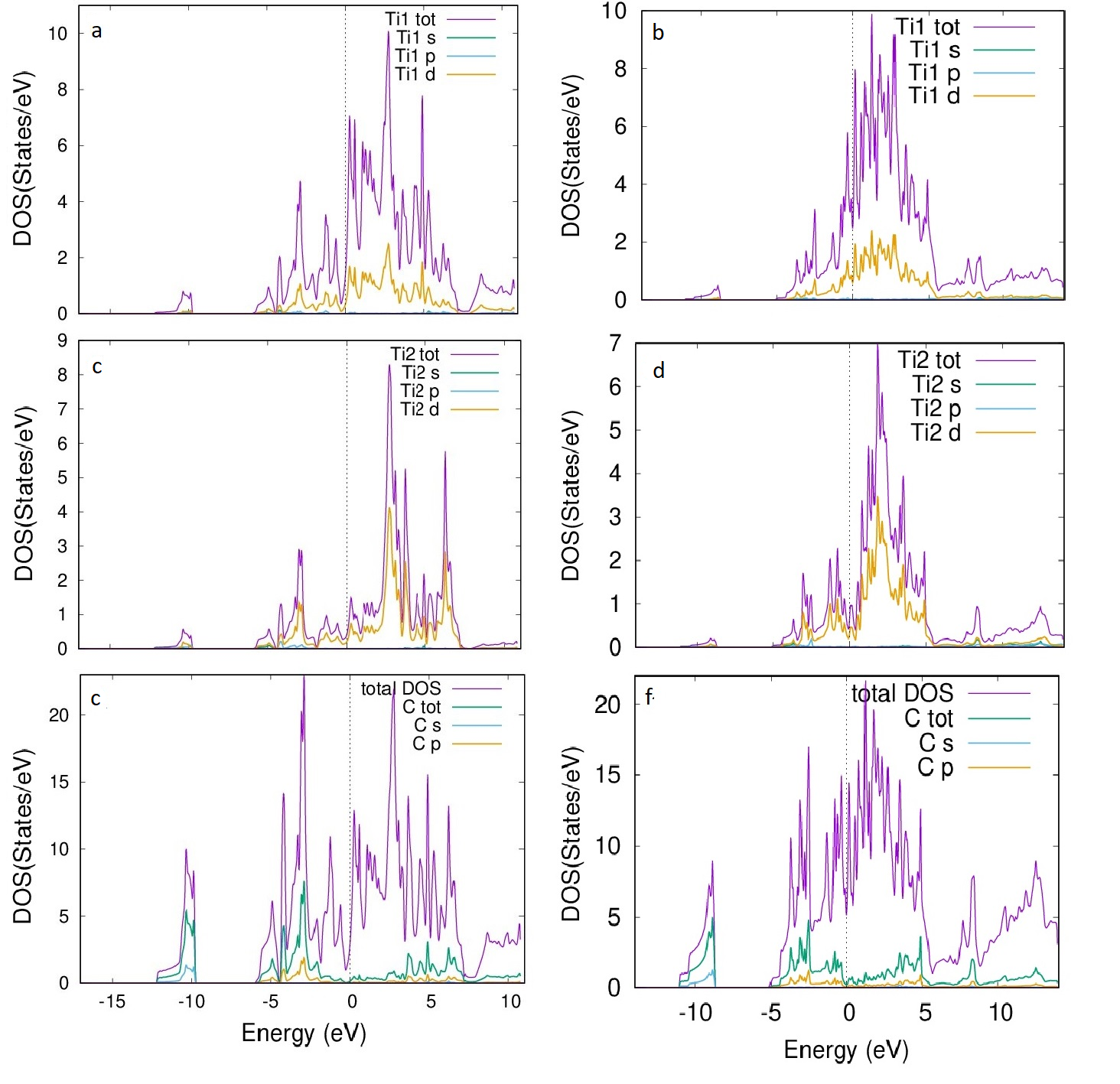


**Figure S4**  Total Partial Density of States for Ti1 Ti2 and C for (a-c) relaxed (d-f) experimental structure of Ti_3_C_2_


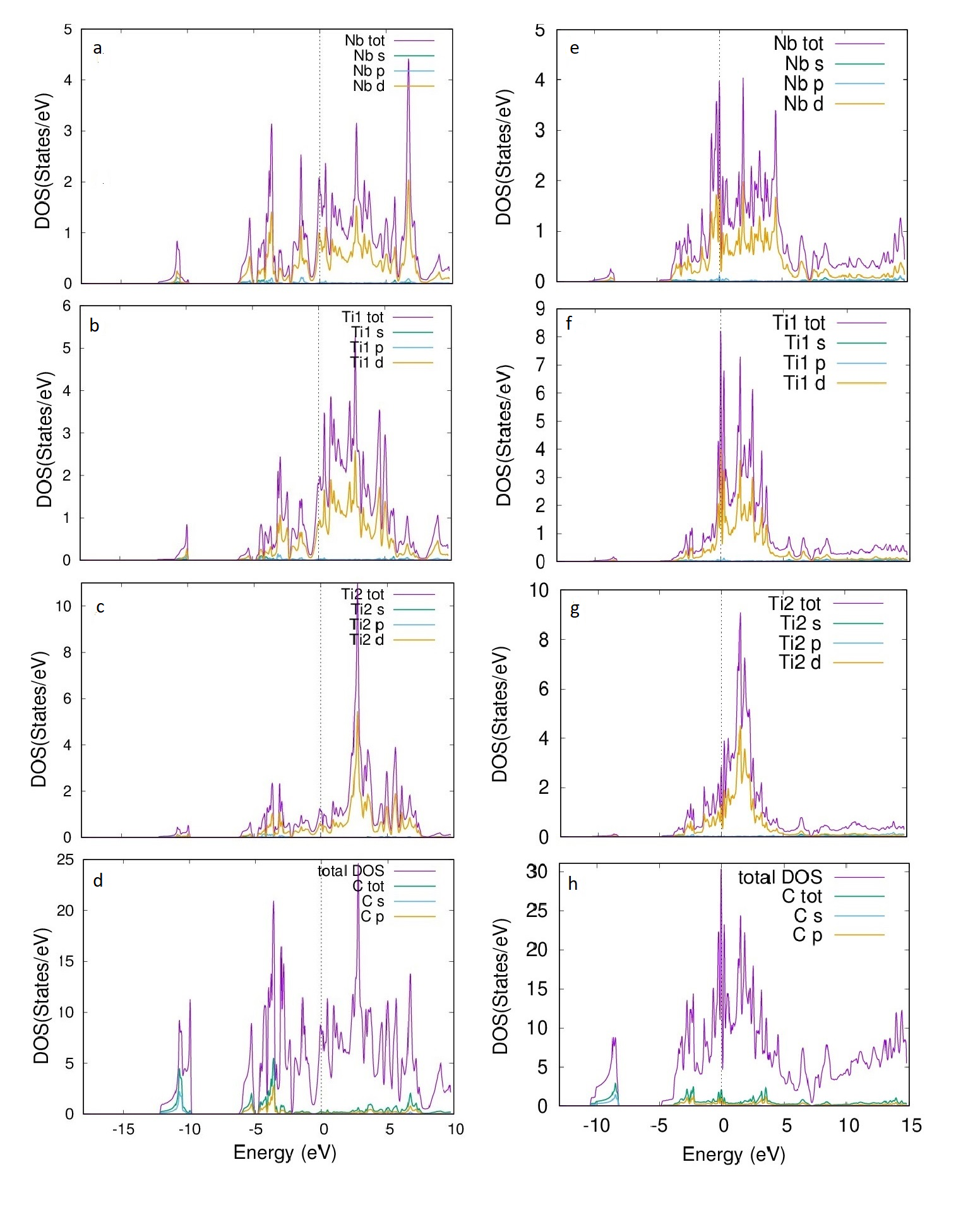


**Figure S5** Total and Partial Density of States for Nb, Ti1, Ti2 and C for (a-d) relaxed (e-h) experimental structure of NbTi_2_C_2_
